# Dual Sensory Loss and Its Mental Health Impacts: Where to Now?

**DOI:** 10.3389/fnagi.2014.00348

**Published:** 2015-01-19

**Authors:** Chyrisse Heine

**Affiliations:** ^1^Department of Human Communication Sciences, School of Allied Health, La Trobe University, Melbourne, VIC, Australia

**Keywords:** dual sensory loss, older adults, mental health, clinical practice, multidisciplinary approach

It is common for older people to experience deterioration of their vision or hearing as they age. The combined effect of vision and hearing loss [known as Dual Sensory Loss (DSL)] is prevalent in the older adult population, occurring in up to 69% of adults aged 65 years and over (Wittich et al., 2012). Concomitant with this impairment is the risk of diminishing physical and mental health (Kiely et al., 2013), decreased communication interactions (Heine and Browning, 2002), and social isolation (Brennan et al., 2006). As eluded in the Heine and Browning (2002) article, in people with DSL, mental health consequences including depression, anxiety disorders, and cognitive aging have not been thoroughly and systematically investigated contributing to a sparse literature on which to base clinical practice.

The issues that arise are: what barriers impede early diagnosis and management of DSL and its mental health impacts, and how are these conditions identified and managed in clinical practice?

The identification of DSL is complicated especially since older adults often acquire this disorder slowly as they age, making DSL difficult to detect particularly in its mild form in either or both of the sensory domains. The research also supports the notion that older people with unisensory loss and those with DSL are not affected by the same impacts, and may not seek or need the same assistance especially if DSL is not identified at the time that unisensory loss is identified. McDonnall (2009) investigated the effect of DSL on depressive symptoms and whether people with DSL were more likely than those with a single sensory loss to experience depressive symptoms. The results of this study suggested that older adults with DSL were likely to experience symptoms of depression similar to those participants with vision loss, but significantly more likely to experience symptoms of depression than participants with hearing loss only. The impacts of unisensory loss and DSL also vary over time. Outcomes of a longitudinal study conducted by Brennan et al. (2006), suggested that in older adults, at baseline, DSL was associated with higher levels of functional disability compared to those adults with unisensory loss; however, the effect gradually diminished over time. These studies highlight the need for professionals to be vigilant in the detection of DSL and not overlook the possibility of an older adult having DSL even if they present symptoms in only one of the domains of vision or hearing loss.

For older adults themselves, numerous barriers to seeking assistance for their sensory losses may exist. It is common for older adults (especially those with DSL) to experience a range of physical and mental health conditions as they age. For example, Crews and Campbell (2004) found that older people with DSL are 2.4 times more likely to report heart disease, 3.6 times more likely to have reported a stroke, and 2.7 times more likely to report depression. It is thus possible that for older adults with DSL, their sensory loss may take on less importance as a priority compared to the other health conditions that may exist co-morbidly. For those with DSL, examples of further barriers preventing health seeking behaviors may include reduced mobility and independence and increased depression (Crews and Campbell, 2004; Brennan et al., 2006).

Vision and hearing health services are usually one-dimensional and the vision or hearing service provider may not be aware of the need for multidisciplinary collaboration. Although separate vision and hearing screening guidelines exist in the US (see U.S. Preventive Services Task Force, 2009, 2012), these are not necessarily adopted in other countries, do not cover DSL, and do not include screening for possible impacts of DSL such as mental health. One example of a multidimensional approach is the Joint Commissioning Strategy for People with Sensory Impairment 2011–2015 proposed by the Surrey County Council, UK^1^. This strategy includes increasing awareness of DSL, ensuring services for this population group meet their complex needs (for example, older adults with DSL and dementia) and that suitable services are offered for those with DSL.

Since DSL and its impacts cross numerous domains, the pathway for service provision is unclear. Professionals are thus encouraged to extend their skills to be able to screen for both sensory losses and identify associated impacts (such as depression and social isolation) so that appropriate referrals can be made. Multidisciplinary collaboration in the management process is necessary so that the best outcome can be achieved for those with DSL (Heine et al., 2002).

In order to encourage optimal mental health, successful aging and sustain quality of life for older adults with DSL, appropriate identification and effective management of older people with DSL is essential. In this regard, a number of points are highlighted including lack of credible and representative research investigating the impacts of DSL on mental health, guidelines regarding pathways for diagnosis and management of DSL in general and more specifically in those with mental health issues, and collaboration among the numerous professionals working in this field including medical and allied health professionals such as medical practitioners and specialists, audiologists, optometrists, and psychologists.

## Conflict of Interest Statement

The author declares that the research was conducted in the absence of any commercial or financial relationships that could be construed as a potential conflict of interest.

## Footnotes

^1^https://www.surreycc.gov.uk/__data/assets/pdf_file/0004/482557/Summary-version-Sensory-impairment-commissioning-strategy-v1.pdf
